# Divergence of environmental filtering: rock exposure rate filters reproductive traits whereas slope shape community richness

**DOI:** 10.3389/fpls.2026.1921286

**Published:** 2026-09-03

**Authors:** Manfu Hou, Xiaoya Yu, Qaiomeng Li, Yili Guo

**Affiliations:** 1 Guangxi Key Laboratory of Environmental Processes and Remediation in Ecologically Fragile Regions, Guangxi Normal University, Guilin, China; 2 Key Laboratory of Ecology of Rare and Endangered Species and Environmental Protection (Guangxi Normal University), Ministry of Education, Guilin, China; 3 College of Environment and Resources, Guangxi Normal University, Guilin, China; 4 School of Tourism and Resource Environment, Qiannan Normal University for Nationalities, Duyun, China; 5 Guangxi Normal University Library, Guilin, China

**Keywords:** community assembly, environmental filtering, pathway divergence, reproductive traits, species richness

## Abstract

**Introduction:**

Environmental filtering is a core mechanism driving plant functional trait reassembly and community succession. However, it is often treated as a uniform process, and whether distinct abiotic factors act through divergent mechanisms remains poorly understood.

**Methods:**

We quantified reproductive traits (fruit types and seed dispersal modes), environmental gradients, and community attributes across plots in a typical resource-limited ecosystem characterized by intensified environmental filtering. Using an integrated framework that combined correlation screening, mediation analysis, and partial correlation confirmation, we identified key environmental factors and disentangled their divergent pathways underlying community richness and reproductive trait composition.

**Results:**

Rock exposure was directly associated with reproductive traits, showing consistent negative effects on nut proportion and consistent positive effects on drupe proportion. In contrast, slope was associated with community attributes, corresponding to significantly higher species richness and reproductive trait redundancy but showing no significant association with reproductive trait composition. This pathway divergence corresponds to a decoupling of community richness from reproductive trait composition.

**Discussion:**

Our results indicate that different environmental filters influence community richness and the proportion of reproductive traits through divergent pathways in resource-limited ecosystems, resulting in decoupling between community attributes and functional traits. This pathway divergence broadens the understanding of functional trait reassembly along multidimensional gradients, and provides novel insights for vegetation restoration in extreme habitats.

## 1 Introduction

Environmental filtering is recognized as a key driver of functional trait reassembly and community succession (Götzenberger et al., 2012). By selecting species with particular traits from the regional pool (Keddy, 1992), environmental filtering generates predictable community patterns along environmental gradients (Sommer et al., 2014; Menezes et al., 2020; Hu et al., 2023). However, environmental filtering has often been conceptualized as a homogeneous process, implicitly assuming that different environmental factors shape communities through similar mechanisms. This assumption overlooks the critical possibility that distinct environmental factors may diverge in their filtering mechanisms, which remains poorly understood. Clarifying such divergent patterns and their underlying mechanisms is essential for our understanding of how environmental filtering shapes community assembly.

Early work focused on whether abiotic filtering or biotic competition dominated assembly, typically using null models to compare observed and random communities (Weiher and Keddy, 1995; Götzenberger et al., 2012). Later studies incorporated more environmental factors and advanced methods (e.g., Elo et al., 2023; Boisseaux et al., 2025), shifting toward quantifying the relative importance of environmental filtering, biotic competition, and dispersal limitation (Kraft et al., 2015; Vellend, 2016). Recent evidence shows that specific factors, such as soil nutrients, water availability, and topography, can independently filter particular functional traits, thereby driving community assembly (Ohdo and Takahashi, 2020; Chen et al., 2023; Sun et al., 2025). However, most studies still aggregate multiple environmental variables into a composite index to assess overall effects, making it difficult to isolate the independent pathways of different factors, let alone test whether their mechanisms diverge (Douce et al., 2023). There is a growing call to move beyond aggregated effects and directly test mechanistic divergence among environmental filters (Gu et al., 2026).

Studies across spatial scales have consistently shown that environmental filtering is the primary driver of community assembly at large scales (e.g., > 30 m; Yuan et al., 2016; Bañares-de-Dios et al., 2020; Mod et al., 2020). As the spatial scale decreases, competition for resources intensifies, and the importance of biotic interactions increases relative to environmental filtering (Bañares-de-Dios et al., 2020; Xu et al., 2021). However, this scale-dependent pattern is not absolute. Strong environmental filtering can also be observed at fine scales (e.g., from a few meters to < 10 m) under conditions of resource limitation or extreme stress (Lhotsky et al., 2016; Kermavnar and Kutnar, 2020; Ortiz et al., 2026). Similarly, earlier successional stages with harsher abiotic conditions consistently exhibit a pronounced environmental filtering effect (Martínez-Ramos et al., 2021). These findings collectively support a general principle: When the intensity of physical stress exceeds the influence of biotic interactions, environmental factors exert stronger filtering pressure on functional traits (Menezes et al., 2020; Hu et al., 2023). Consequently, systems with highly restricted resources offer a unique opportunity to isolate and identify how different environmental factors shape community assembly and functional trait composition.

Reproductive traits (e.g., fruit type, seed dispersal mode) are key life-history characteristics that directly determine community regeneration and ecosystem resilience (Howe and Smallwood, 1982; Beckman and Sullivan, 2023), and have received increasingly attention in recent years (Larson and Funk, 2016; Carmona and Beccari, 2025; Silveira et al., 2026). Substantial evidence confirms that the reassembly of reproductive traits is driven primarily by environmental filtering (Díaz et al., 1998; Chen et al., 2017; Han et al., 2019; MacTavish and Anderson, 2020; Acosta-Rojas et al., 2023). Animal-mediated dispersal, especially of fleshy fruits, predominates in species-rich habitats with abundant animal vectors (Vasconcelos et al., 2023; Beckman and Sullivan, 2023), whereas dispersal strategies independent of biotic vectors (e.g., dry fruits and autochory) are favored in resource-limited or highly disturbed environments (Ramírez et al., 2021). This shift in trait composition can arise either from direct environmental filtering or indirectly through species turnover or diversity changes (Lasky et al., 2014; Mittelbach and Schemske, 2015; Hua et al., 2024). However, it remains unclear whether different environmental factors influence reproductive traits through divergent mechanistic pathways and how such potential divergence modulates the coupling between community assembly and functional trait assembly.

The subtropical karst region of southern China represents a classic resource-limited ecosystem. Characterized by extensive bedrock exposure, precipitous slopes, patchy and shallow soils, and recurrent geological droughts, this ecosystem creates a steep composite gradient of globally extreme physical stress and high spatial heterogeneity (Jiang et al., 2014; Hu et al., 2023; Li et al., 2025). This makes it an ideal natural laboratory globally for disentangling how distinct abiotic factors shape environmental filtering patterns. In this study, we integrated correlation screening, mediation analysis, and partial correlation confirmation to investigate the relationships among reproductive trait variation, environmental factors, and community attributes in this typical resource-limited ecosystem. We hypothesized that (1) intense substrate limitation is directly associated with reproductive trait assembly through environmental filtering in this highly heterogeneous and resource-limited system. (2) different abiotic factors may influence reproductive trait assembly through divergent pathways. By testing these hypotheses in a globally representative resource-limited ecosystem, this study provides empirical insights into functional trait reassembly through divergent rather than uniform environmental filtering patterns.

## 2 Materials and methods

### 2.1 Study site

The study was conducted in a nature reserve (103.97° - 104.55° E, 24.38° - 24.75° N; 1,800 to 2,100 m elevation) within the core zone of the South China Karst region. The area has a subtropical monsoon climate with a mean annual temperature of 15.8 °C and mean annual precipitation of 1,235.6 mm. The bedrock is primarily pure carbonate rock, which experiences intense weathering to form shallow, discontinuous soils that impose strong nutrient and water stress. The vegetation is dominated by evergreen and deciduous broad-leaved mixed forest, interspersed with patches of deciduous broad-leaved forest (He et al., 2021). The primary disturbances are grazing and light logging by adjacent villages, creating a disturbance gradient that declines with distance and relative elevation from the villages. These intersecting natural and anthropogenic gradients generate the environmental heterogeneity and successional variation examined in this study.

### 2.2 Plot survey and determination of reproductive traits

Plot surveys were conducted in June 2018 and August 2019 along topographic and anthropogenic disturbance gradients within the study area following Fang et al. (2009). Environmental variables and community attributes were recorded for each plot. Slope aspect and slope angle were measured with a portable 3D electronic geological compass (DZL-1G, Harbin Optical Instrument Factory, China). Slope angle ranged from 6° to 41° (mean = 19°), while slope aspect was recorded in degrees clockwise from north (0°–360°) and subsequently converted to a continuous variable via a cosine transformation (cos(*aspect*)). Elevation and coordinates of each plot and village were recorded using a handheld GPS receiver (A8, Hefei Zhuolin Technology Co., Ltd, China). Disturbance of the plots was based on their minimal distances to village (DV) and relative elevation difference to village (EDV) from the villages around. The rock exposure rate (RER) was visually estimated (coverage 10-98%). Slope position was also visually estimated and then converted to ordinal grade values in ascending order (e.g., hilltop = 1, lower slope = 7, see supplementary **Supplementary Table 1**).

Species richness was measured as the number of species per plot. Reproductive trait redundancy (RTR) was defined as the mean number of species per reproductive trait category, which aligns with the classic definition of functional redundancy (Walker, 1992). It was calculated as (Mouillot et al., 2014):


RTR=S/T

where *S* and *T* are, respectively, the total species richness and the number of reproductive trait categories within a plot.

Leaf life form composition was represented by the importance value of either evergreen or deciduous tree species; here we used that of deciduous species (deciduous IV). Importance value (IV) was calculated following Fang et al. (2009):


IV(tree layer) = (relative dominance + relative abundance + relative frequency)/3

IV(shrub/herb layer) = (relative height + relative coverage + relative frequency)/3

Fruit type classification followed the fruit type system of Spjut (1994). Fruit types of species were determined according to the *Flora Reipublicae Popularis Sinicae* (Editorial Committee of FRPS, 1959–2004) and published literature. A functional approach was adopted in two cases: the fruit of *Juglans mandshurica* was classified as a nut rather than a drupe, and the aggregate achenes of *Fragaria nilgerrensis* were treated as fleshy fruits. Furthermore, to include all vascular plant species, fern spores were considered a functional fruit type (Blake-Mahmud et al., 2025). Seed dispersal modes were assigned using the Royal Botanic Gardens Seed Information Database, Kew (https://ser-sid.org/) and published sources (Yu and Li, 2019; Lososová et al., 2023; Wang et al., 2025). For species with unidentified dispersal modes from these sources, it was inferred from their fruit traits and the dispersal mechanism of congeneric species (Guo and Zheng, 2017; Yu and Li, 2019). All data are available in **Supplementary Table 1**.

### 2.3 Calculation of the success degree index

The degree of succession was proposed by Numata (1969) to quantify the developmental degree of a community, and has since been refined and adopted by other ecologists (Zhang, 1994). It is based on static indices derived from species’ life-history traits and reflects the substrate constraints and topographic heterogeneity on community assembly. The formula is as follows (Zhang, 1994):


$$D=\left(\overset{p}{\underset{i=1}{\sum }}I_{\text{i}}\times d_{\text{i}}/N\right)\times V$$

where *D* represents the successional degree index, *I_i_* is the longevity score for species *i* (annual = 1, biennial = 2, chamaephyte/geophyte = 10, large shrub/small tree = 50, medium/large tree = 100), *d_i_* is the dominance of species *i* expressed as its importance value (IV), *N* is the total number of species in the plot and *V* is the vegetation cover.

### 2.4 Correlation analysis and variable screening

Twenty-six variables were included for analysis: 6 environmental factors (RER; slope; slope position; aspect; DV; EDV), 3 community attributes (species richness; deciduous IV; RTR), and 17 reproductive traits (13 fruit types and their aggregated dry fruit proportion; 3 dispersal modes; see **Supplementary Table 1**). Given the large number of traits relative to sample size, identifying those most ecologically informative before formal hypothesis testing is necessary. We employed a systematic correlation-guided screening procedure followed by exploratory mediation and partial correlation analyses.

An exploratory Spearman correlation heatmap was generated using raw values to examine overall association patterns and identify potential multicollinearity among variables. To identify correlations among key variables, we selected variables based on both ecological function and statistical importance. All environmental factors and community attributes were included as predictor variables based on their ecological relevance. The successional degree index was excluded to avoid circular reasoning. Reproductive traits were retained based on the number of significant associations after false discovery rate (FDR) correction (Benjamini and Hochberg, 1995) and the sum of their absolute correlation coefficients (|*ρ*|). This procedure identified nut and drupe as the representative fruit types, because they had the highest number of significant associations, the largest sum of |*ρ*|, and were represented in all or the vast majority of plots, whereas follicle was excluded due to its absence in 25% of plots (see **Supplementary Tables 1**, **S2**; **Supplementary Figure 1**). Nut well represented the dry fruit strategy, while drupe, the fleshy strategy. Additionally, to comprehensively evaluate reproductive strategies, zoochory was retained as a core reproductive response variable because it is the dominant seed dispersal mode (representing 62% of the total; see **Supplementary Table 1**), thereby allowing the environmental filtering effects on this dispersal strategy to be explicitly tested.

The multicollinearity among the final 12 core variables was evaluated using the variance inflation factor (VIF, **Supplementary Table 3**). Following the widely accepted criterion proposed by Hair et al. (2010), a VIF< 10 indicates an acceptable level of collinearity that does not substantially affect coefficient estimation. Only richness and RTR showed collinearity (VIF > 10), yet both were retained in the main analyses and designated as candidates for removal in sensitivity analyses in mediation models. Finally, a correlation network was constructed using the 12 core variables, including only those associations that were significant after FDR correction (adjusted *p*< 0.05) and had |*ρ*| > 0.3.

### 2.5 Piecewise mediation analysis for potential filtering pathways

To identify the pathways of association among environmental gradients, community attributes, and reproductive traits, we used piecewise mediation analysis (Grace et al., 2010; Lefcheck, 2016). Based on the 12 core variables identified from correlation network analysis and following the hierarchical filter framework of community assembly theory (Keddy, 1992), the environmental gradient was specified as the initial driver (*X*), community attributes as potential mediators (*M*), and reproductive traits as response variables (*Y*).

All variables were standardized (mean = 0, standard deviation = 1) and path effects were reported as standardized coefficients. The direct effect was defined as the net effect (standardized regression coefficient *β*) of *X* on *Y* after controlling for all *M* and other predictors. The indirect effect was calculated as *a* × *b*, where *a* is the effect of *X* → *M* and *b* is the effect of *M* → *Y* (Grace et al., 2010). A random seed (2025) was used for all bootstrap analyses within a single workflow to ensure reproducibility. To estimate bias-corrected and accelerated accelerated bootstrap confidence intervals (BCa CI; Efron, 1987), 5000 bootstrap resamples were performed. The paths were considered statistically significant if their 95% CI did not include zero. To ensure the reliability of the BCa interval estimates, the skewness coefficient (|*skewness*|< 2 considered acceptable) and the coefficient of variation (*CV* ≤ 1.0 considered stable, Preacher and Hayes, 2008) were used to test the bootstrap sampling distribution (**Supplementary Table 4**).

To evaluate the robustness of the identified pathways, multi-model sensitivity analyses were performed (Lefcheck, 2016). These included: the Full model (all 12 variables), the NoAspect model (excluding aspect, which showed no significant association with response variables in the correlation network), the NoRTR model (excluding RTR), and the NoRichness model (excluding species richness, which was highly collinear with RTR; variance inflation factor, VIF = 18.87 for richness in the Full model, see **Supplementary Table 3**). Based on the significance patterns across these models, the pathways were categorized into four types: (1) consistent, significant in the Full model and in at least one sensitivity model; (2) context dependent, significant only in the Full model; (3) conditional, not significant in the Full model but significant in two or more sensitivity models; (4) unstable, significant in only a single sensitivity model.

### 2.6 Partial correlation assessment of stable pathways

To confirm the stability of the mediation-identified potential pathways under an alternative analytical framework, partial correlation analysis was conducted to test whether significant path segments retained stable associations after controlling for theoretically relevant variables. The control variables were defined according to path type. For *X* → *M* path segments, all other environmental variables were controlled to test the unique effect of a specific environmental factor on community attributes. For *M* → *Y* path segments, all environmental variables were controlled to capture the collective influence of community attributes on reproductive traits while preserving their natural ecological links. For direct *X* → *Y* paths, all community attributes were controlled to exclude their potential mediating effects. The significance of partial correlation coefficients was determined using 95% BCa bootstrap confidence intervals (as described in Section 2.5). A potential pathway was considered a consistent and ecologically meaningful association only if its corresponding path segments were significant in the partial correlation analysis.

## 3 Results

### 3.1 Patterns of association among environment, community, and reproductive traits during forest succession

**Figure 1:**
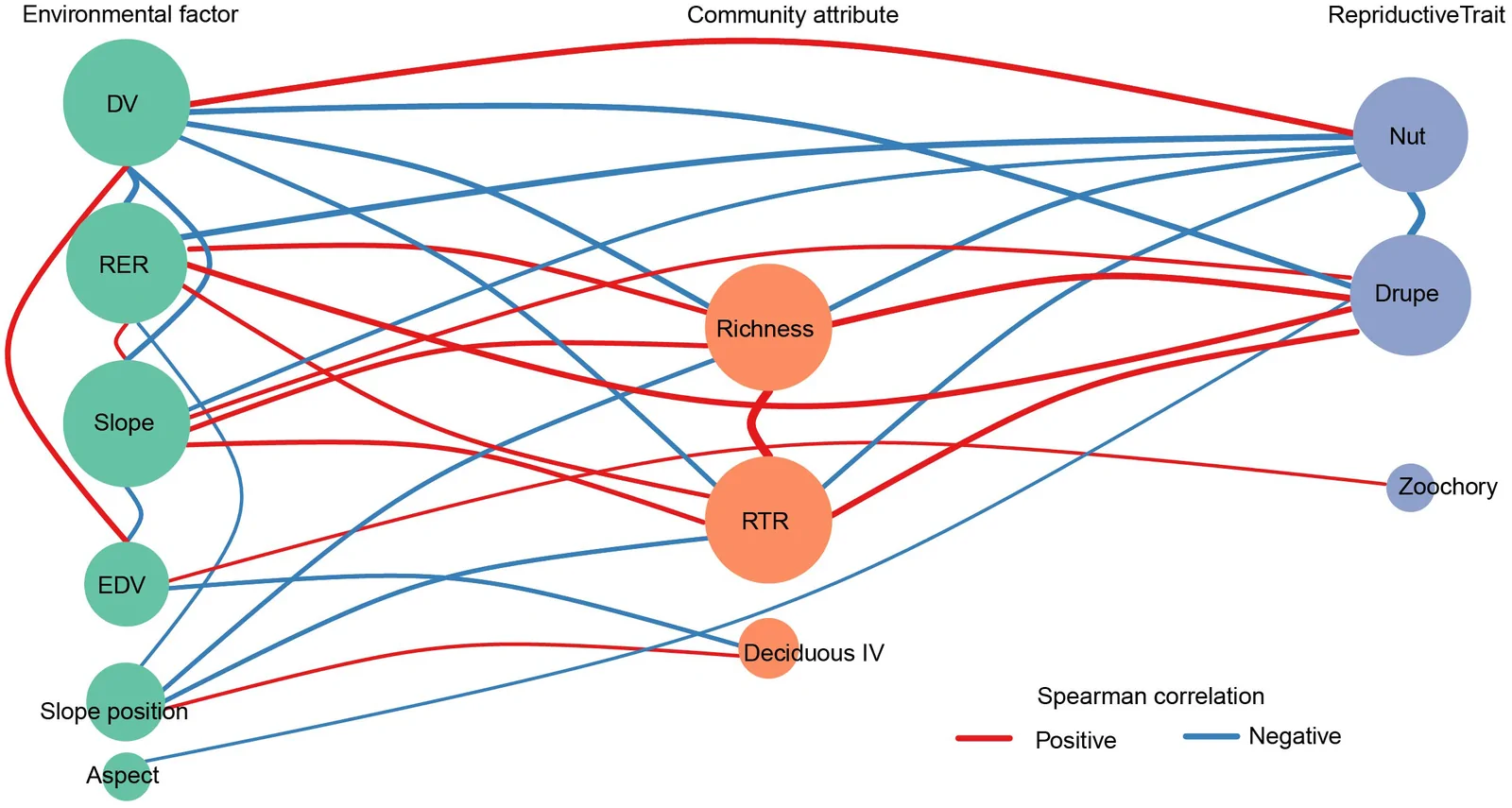
Spearman correlation network among major factors of the resource-limited forest ecosystem. The edges represent significant correlations (p < 0.05) with |ρ| > 0.3, where ρ is the Spearman correlation coefficient, and edge width is proportional to |ρ|. Note: RER, rock exposure rate; DV, distance to the village, indicates intensity of human disturbance; EDV, elevation difference from the village, RTR, reproductive trait redundancy; Deciduous IV, important value of deciduous tree species.

The Spearman correlation analysis revealed extensive associations among environmental factors, community attributes, and reproductive traits in the forest (**Supplementary Figure 1**). Variables meeting the criteria of FDR-corrected *q*< 0.05 and |*ρ*| > 0.3 formed a tightly correlated network (**Figure 1**; **Supplementary Table 5**), identifying the core factors within environmental factors (DV, RER, and slope), community attributes (richness and RTR), and reproductive traits (proportions of nuts and drupes). Strong and systematically opposing patterns emerged among these key variables. For example, both RER and slope showed significant positive correlations with richness, RTR, and drupe proportion, but significant negative correlations with nut proportion. Conversely, DV was significantly negatively correlated with richness, RTR, and drupe proportion, but significantly positively correlated with nut proportion. In contrast, the associations involving aspect, deciduous IV, EDV, slope position, and zoochory were sparse or weak.

### 3.2 Potential pathways of environmental gradients on reproductive traits

**Figure 2:**
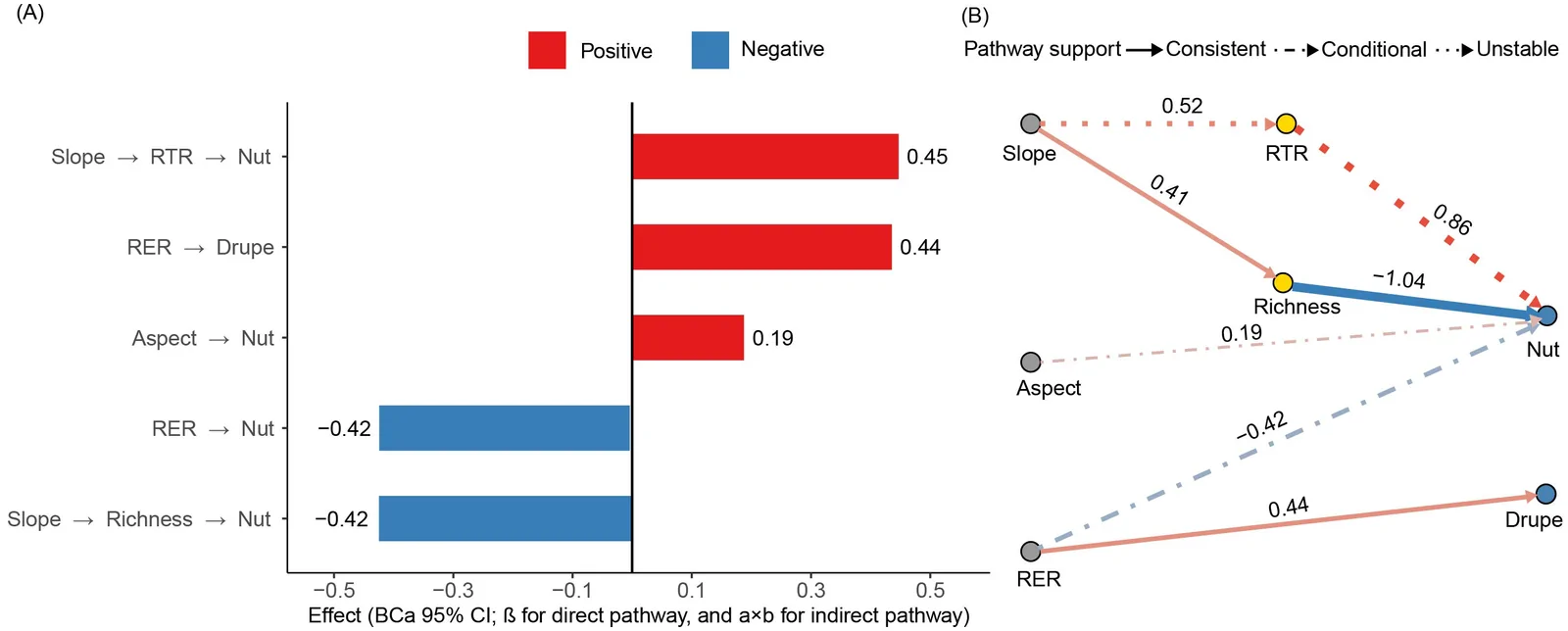
Standardized effects of significant pathways identified by mediation analysis **(A)** and their mechanism network **(B)**. The colors of the lines indicate the direction of the effect (red = positive, blue = negative), and the width of the line is proportional to |β|. RER, rock exposure rate; RTR, reproductive trait redundancy. The line type of Consistent, significant in the Full model and in at least one sensitivity model; Conditional, not significant in the Full model but significant in two or more sensitivity models; Unstable, significant in only a single sensitivity model.

The mediation analysis identified five significant pathways, comprising two consistent, two conditional, and one unstable pathway (**Figure 2A**; **Supplementary Tables 4**, **S6**). The slope showed an indirect association with nut proportion through two community attributes. Steeper slopes were negatively associated with nut proportion through increased species richness (effect = –0.42, 95% CI: [-1.31, -0.005]; **Figure 2B**; **Supplementary Table 6**). This indirect effect was significant in the full model and NoAspect model, but became nonsignificant in the NoRTR model (**Supplementary Table 6**). In contrast, RER showed a direct positive association with drupe proportion (0.44, CI: [0.01, 1.18]), and was significant across all three sensitivity models.

RER and slope aspect both showed conditional direct positive association with nut proportion. They were not significant in the Full model (–0.42, CI: [-0.92, 0.004]; 0.19, CI: [-0.04, 0.46]), but both became significant across sensitivity models (**Supplementary Table 6**). In contrast, the unstable pathway (Slope → RTR → Nut) was significant only in the NoAspect model (0.45, CI: [0.02, 1.27]; **Supplementary Table 6**). Key associations remained consistent in sensitivity models excluding either richness or RTR, indicating the stability of mediation analysis despite initial multicollinearity.

### 3.3 Partial correlation confirmation of pathway stability

**Figure 3:**
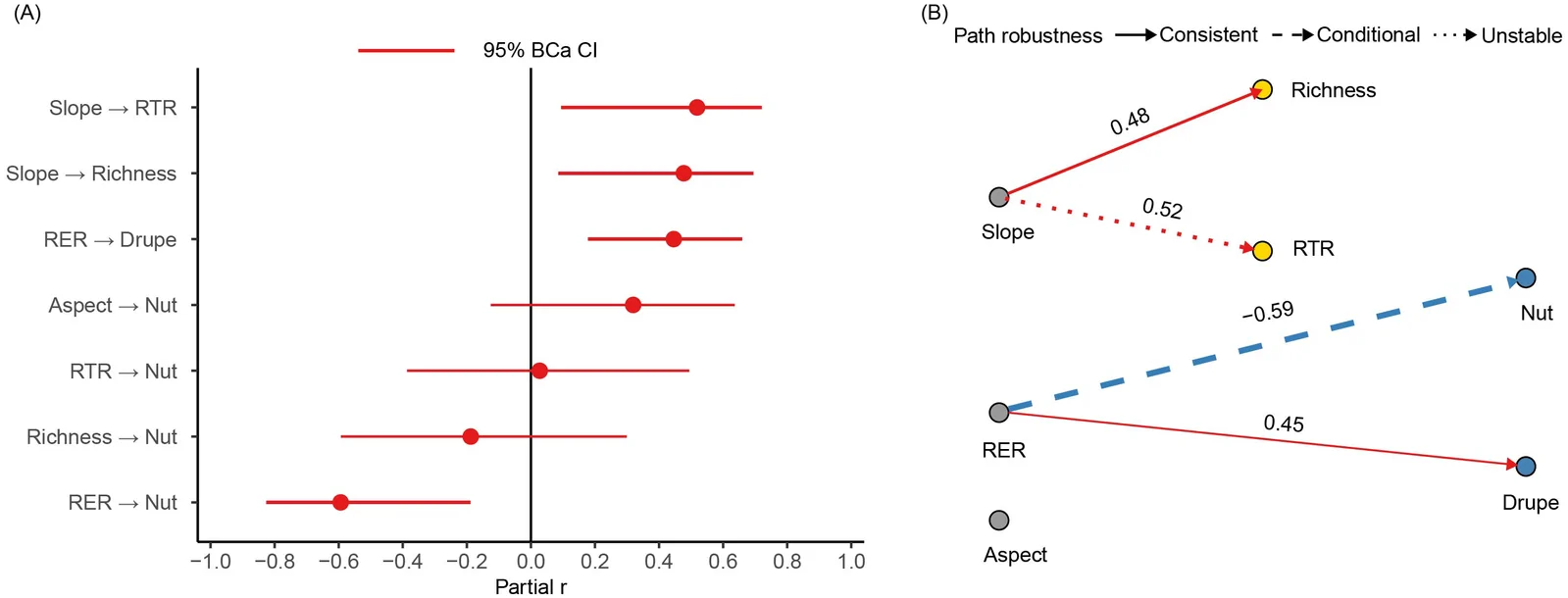
Partial correlation validation of segmentation of the pathways from mediation analysis **(A)** and network of the four significant pathways **(B)**. Thick lines indicate significant associations (95% BCa CI excludes zero). RER, rock exposure rate; RTR, reproductive trait redundancy. The line type of Consistent, significant in the Full model and in at least one sensitivity model; Conditional, not significant in the Full model but significant in two or more sensitivity models; Unstable, significant in only a single sensitivity model.

Partial correlation analysis revealed that only a subset of the pathways identified by the mediation analysis remained significant (**Figure 3A**; **Supplementary Table 7**; effect decomposition verified for computational accuracy in **Supplementary Table 8**). For the consistent indirect pathway (Slope → Richness → Nut), only the first segment (Slope → Richness) withstood screening (*r* = 0.48, CI: [0.09, 0.69]); the second segment (Richness → Nut) did not withstand screening (*r* = -0.19, CI: [-0.59, 0.30]). The unstable indirect pathway (Slope → RTR → Nut) showed the same pattern: the first segment (Slope → RTR) remained significant (*r* = 0.52, CI: [0.09, 0.72]), whereas the second segment (RTR → Nut) did not (*r* = 0.03, CI: [-0.39, 0.49]), indicating that both indirect pathways were interrupted rather than fully mediated. By contrast, two direct pathways withstood screening: the consistent positive pathway from RER to drupe proportion (RER → Drupe: *r* = 0.45, CI: [0.18, 0.66]) and the conditional negative pathway from RER to nut proportion (RER → Nut: *r* = -0.59, CI: [-0.83, -0.19]). The other conditional direct pathway (Aspect → Nut) failed to withstand screening (*r* = 0.32, CI: [-0.13, 0.64]). Overall, only two direct pathways and the first segments of both indirect pathways passed the partial correlation screening (**Figure 3B**). The significance of several conditional and unstable pathways in mediation analysis demonstrates that the sensitive models effectively uncover complex associative patterns obscured by multicollinearity.

## 4 Discussion

### 4.1 Rock exposure rate is strongly associated with shifts in reproductive traits

Our results are consistent with a strong association between RER and shifts in reproductive trait proportions, corresponding to a higher proportion of drupes and a lower proportion of nuts, which may reflect a trade-off between the two reproductive strategies. This supports the view that resource limitation intensifies environmental filtering (Menezes et al., 2020; Hu et al., 2023). In subtropical karst forests, the solubility of carbonate bedrock results in slow pedogenesis and shallow, discontinuous soils with low total soil mass (Wang et al., 1999). Furthermore, the bedrock contains an extensive network of fissures and solution-enlarged conduits (Yuan et al., 1991; Ford and Williams, 2007). This allows water and soil to be lost not only from the surface but also vertically through these fissures and conduits, further reducing water and nutrient availability and intensifying environmental stress (Chen et al., 2024; Dai and Peng, 2025). By imposing physiological limits on species, this physical constraint contributes to adjustments in resource allocation strategies (Krebs, 2014) and may reinforce direct environmental filtering. As a comprehensive resource index, RER effectively captures physical conditions including soil cover and continuity, water and nutrient availability, and microhabitat stability that impose hard constraints on seed germination and seedling establishment (Shen et al., 2018). Consequently, in habitats with highly exposed bedrock and scarce soil, plants tend to invest in reproductive strategies that enhance colonization assurance while reducing investment in riskier ones (Lei et al., 2012; Beckman and Sullivan, 2023).

This differential filtering may reflect the divergence of plant adaptive strategies under resource-limited karst ecosystems. The drupe strategy attracts a diverse array of vectors via its fleshy mesocarp while relying on a hard endocarp to shield the seed, thereby reducing dispersal and establishment risks in habitats with high bedrock exposure (Howe and Smallwood, 1982). In contrast, the nut strategy is highly dependent on stable animal dispersal networks and specific microhabitats, conditions that are often scarce in these extreme physical environments (Lei et al., 2012; Beckman and Sullivan, 2023). This may lead to the dominance of drupe species and a marked competitive disadvantage of nut species in such high RER areas. Previous studies on the effects of karst substrate limitation have primarily focused on leaf economic spectrum traits or general functional traits (Hu et al., 2023; Beccari and Carmona, 2024). Some studies have observed impacts on reproductive strategies but did not quantify their differential responses (Li et al., 2021; Hu et al., 2023). Our findings fill this gap by clearly distinguishing, for the first time in a natural system, the divergent environmental filters effects of RER on different reproductive strategies.

The association between RER and reproductive traits suggests that physical substrate constraints shape plant strategies (Menezes et al., 2020). By using RER as a composite resource index, our study identified its stronger predictive power for reproductive traits in resource-limited environment. Furthermore, this filtering effect is closely linked to substrate limitation. It is independent of co-varying climatic or nutrient gradients (Hu et al., 2023) and remains consistent to topographic and community-level variations. These findings provide a valuable case study for understanding the adaptive evolution of reproductive traits under extreme physical stress.

### 4.2 Divergence in environmental filtering pathways

Our results reveal that slope significantly corresponds to increased community richness (including both species richness and reproductive trait richness), but this influence does not effectively extend to reproductive trait composition. Unlike RER, which comprehensively represents resource availability, slope primarily modifies habitat quality indirectly through the redistribution of water, nutrients, and heat. Steep slopes increase the risk of soil erosion, subsequently increasing RER, and thereby reducing the overall supply of soil nutrients and water (Chen et al., 2024), creating stronger environmental stress that can reinforce environmental filtering (Menezes et al., 2020; Hu et al., 2023). However, the intense soil and water movement on steep slopes simultaneously leads to soil accumulation at the upslope microsites of exposed bedrock, where nutrients and water are retained, while causing losses at the downslope counterparts, thereby increasing spatial heterogeneity and resource niches (Chen et al., 2022). Such spatial patterns may promote species coexistence and explain, at least within a moderate range of slopes (e. g., most< 30° in this study; see **Supplementary Table 1**), why community richness increases with slope gradient (Lhotsky et al., 2016; Pang et al., 2025).

The increase in species richness did not systematically alter the relative composition of reproductive traits (e.g., the ratio of nuts to drupes), contrary to the previous expectation that the increase in richness would accompany the functional trait diversity (Lasky et al., 2014). This dissociation of the pathways suggests a crucial insight: the influence of environmental factors on community attributes does not automatically translate into filtering pressure on functional traits. The underlying reason may arise from the divergent patterns of these two environmental factors. As a composite index of substrate limitation, RER represents a physiological constraint that filters species with specific reproductive traits (Shen et al., 2018). These co-occurring species tend to share similar, stress-tolerant reproductive strategies, leading to high reproductive trait redundancy (Kraft et al., 2015; Hu et al., 2023). In contrast, slope creates resource heterogeneity that relates to species coexistence patterns (Chen et al., 2022; Pang et al., 2025), but operates only within the trait pool pre-filtered by RER. Consequently, an increase in species richness or RTR simply adds functionally redundant species without altering the overall proportion of reproductive traits. Thus, the lack of significant associations between community attributes and trait composition reflects a genuine ecological decoupling (Robroek et al., 2017). In other words, environmental filtering may determine which reproductive strategies can assemble in the community, while micro-habitat heterogeneity regulates how many species can coexist within the pre-filtered pool. Operating at different hierarchical levels, these two processes may ultimately lead to the decoupling of community richness and reproductive trait composition. This hierarchical interpretation represents a plausible ecological hypothesis requiring further validation.

This decoupling may be reinforced by multiple ecological mechanisms. First, co-occurring species within the environmental filtering trait pool tend to share similar reproductive strategies, which may contribute to high reproductive trait redundancy. Consequently, changes in community richness do not necessarily alter the overall proportion of reproductive traits. Second, the conservatism of reproductive traits may lead to lagged responses in community turnover (Peterson, 2011); thus, the currently observed community state may reflect the filtering outcomes from past environmental conditions, and reproductive traits have not yet fully adjusted. Third, detectable changes in reproductive trait composition may become apparent primarily when the community undergoes drastic reorganization (Lasky et al., 2014). Furthermore, the seed dispersal mode (zoochory) did not exhibit significant environmental filtering signals in our findings, representing an additional layer of trait decoupling (fruit types vs. dispersal modes). This suggested that decoupling in extreme resource-limited karst environments may be multifaceted and pronounced. Robroek et al. (2017) demonstrated that community and functional trait assembly could be decoupled. Our study provides empirical evidence for this decoupling and further demonstrates that it may arise under specific ecological contexts.

In summary, in such a physically stressful and highly heterogeneous environment, the pathways of different abiotic factors diverge: RER is directly associated with reproductive traits, whereas slope modulates community structure without altering trait composition. This divergent associative framework, combined with trait redundancy, conservatism, and high response thresholds, may jointly explain why community richness increases while reproductive trait composition remains unchanged. This decoupling suggests that environmental filtering is not a homogeneous process but a heterogeneous process with distinct functional outcomes. It also highlights the inherent complexity of environmental filtering and functional stability, underscoring the necessity of distinguishing the action pathways of different abiotic drivers in systems where multidimensional environmental gradients co-occur.

### 4.3 Ecological implications and limitations

This study provides empirical evidence that distinct environmental factors influence reproductive trait assembly and community richness through divergent pathways in resource-limited karst forests, where trait-based assembly has been shown to be dominated by environmental filtering across forest types (Jiang et al., 2024). All of these pathways are modulated by the combination of resource stress and heterogeneity. Our findings prompt a conceptual shift from viewing environmental filtering as a homogenizing process to recognizing it as a divergent pattern within a refined framework. This framework needs to acknowledge the reinforcement and diversification of environmental filtering by resource limitation and environmental heterogeneity (Lhotsky et al., 2016; Pang et al., 2025; Ortiz et al., 2026). The RER intensifies environmental filtering through physical constraints, while the slope may promote species richness by enhancing heterogeneity (Menezes et al., 2020; Chen et al., 2024; Pang et al., 2025). These two types of factors constitute a multidimensional environmental gradient that underlies pattern divergence and leads to decoupling of community richness and functional traits.

The decoupling of environmental filtering effects on functional traits versus community attributes offers potential management implications for vegetation restoration. Interventions targeting substrate exposure may influence reproductive trait composition, whereas management of topographic factors should be more relevant to maintaining community diversity to support long-term succession processes. Such differentiated management strategies, grounded in pathway divergence, provide testable hypotheses for improving restoration outcomes in vulnerable rocky ecosystems. While based on a specific karst forest system, this case study broadens the understanding of how such pattern differentiation may operate in other resource-constrained environmental gradient systems.

Despite these insights, several limitations and uncertainties should be acknowledged. First, the small sample size (n = 28) and the presence of multicollinearity among predictors constrain the inferential strength of complex models, limiting the detection of weak-effect pathways and increasing the risk of overfitting (Graham, 2003). Although our multi-step workflow mitigates these concerns (e.g., sensitivity analyses were employed mitigate multicollinearity), it cannot entirely eliminate statistical uncertainty. Second, composite indicators such as RER and slope are derived primarily through inference, as specific metrics (e.g., micro-scale soil nutrient content, water content) were not directly monitored. Third, the significance of some pathways varied across sensitivity analyses, indicating that these associations are partially conditional upon model specification. Therefore, the ecological processes discussed herein should be interpreted as plausible working hypotheses consistent with our statistical associations, rather than demonstrated causal mechanisms.

Within these constraints, our study provides testable predictions. Future research should integrate large-scale, multi-plot data to test the generalizability of these divergent filtering patterns across broader spatial scales (Mod et al., 2020). Detailed environmental monitoring including biotic factors such as mycorrhizal fungi that mediate plant resource acquisition, survival, and competitive coexistence, coupled with controlled microcosm experiments or isotopic tracing to quantify resource allocation trade-offs, could more directly test the proposed mechanisms of environmental filtering and their divergence in resource-limited ecosystems (Xu et al., 2021; Douce et al., 2023; Wagg and McKenzie-Gopsill, 2023). Additionally, adopting pre-registered, directed acyclic graph (DAG) based selection frameworks would further validate these filtering divergences (Borger and Ramesh, 2025).

## 5 Conclusion

This study systematically examined how environmental gradients are associated with reproductive traits and community attributes in a typical resource-limited karst ecosystem. Our findings reveal that environmental filtering operates through distinct associative pathways: RER is consistently associated with shifts in reproductive trait composition (corresponding to higher drupe and lower nut proportions), whereas slope is associated with increased species richness independent of changes in trait composition. This decoupling of diversity and functional responses is underpinned by the combined effects of resource limitation and high spatial heterogeneity, highlighting environmental filtering as an inherently heterogeneous process. By disentangling these pathways, our work advances functional trait ecology beyond filtering intensity toward a refined understanding of how abiotic drivers differentially shape communities. These insights provide an empirical basis for vegetation restoration in extreme habitats, where species selection should account for the divergent roles of environmental filters in shaping both species richness and functional composition.

## Data Availability

The datasets presented in this study can be found in online repositories. The names of the repository/repositories and accession number(s) can be found in the article/**Supplementary Material**.
